# Changes in Plasma Metabolites Concentrations in Obese Dogs Supplemented With Anti-oxidant Compound

**DOI:** 10.3389/fnut.2018.00074

**Published:** 2018-09-07

**Authors:** Koh Kawasumi, Tae Murai, Takayuki Mizorogi, Yuki Okada, Ichiro Yamamoto, Kohei Suruga, Kazunari Kadokura, Toshiro Arai

**Affiliations:** ^1^Laboratory of Veterinary Biochemistry, School of Veterinary Medicine, Nippon Veterinary and Life Science University, Musashino, Japan; ^2^Food Function R&D Division, International Operation Department, Kibun Foods Inc., Inagi, Japan

**Keywords:** anti-oxidant, dog, lipid metabolism, liver function, *Rhus verniciflua*

## Abstract

The aim of this study is to discuss the effect of anti-oxidant supplement (Rv-PEM01-99, Kibun Foods, Inc., Tokyo, Japan) on changes in energy metabolism in obese dogs. 200 mg/kg/day of Rv-PEM01-99 (equivalent to 5 mg kg/day of quercetin derivative) were applied for 6 weeks to the Beagle dogs fed high fat diet (HFD) or control diet (CD). In the present study, body weight (BW) decreasing effect of Rv-PEM 01-99 in obese dogs was not clear. However, plasma alkaline phosphatase (ALP) activities at the end of experiment were significantly decreased compared to those at the start of experiment in obese dogs supplemented with Rv-PEM 01-99 (paired-*t* test, *p* < 0.05). In control dogs supplemented with Rv-PEM 01-99, Plasma malondialdehyde (MDA), and triglycerides (TG) levels and lactate dehydrogenase (LDH) activities were significantly decreased compared to those at the start of experiment (paired-*t* test, *p* < 0.05). From these findings, Rv-PEM 01-99 seems to be not harmful for dogs. Anti-lipid peroxide effect and liver function improvement are expected in the dogs supplemented with Rv-PEM 01-99.

## Introduction

Recently, occurrence of obesity and its related metabolic disorders have increased in dogs and cats as in humans (1, 2). Obesity accompanied by visceral fat accumulation causes insulin resistance and is a risk factor for metabolic syndrome, diabetes mellitus, hypertension, dyslipidemia, cardiovascular diseases, musculoskeletal disorders, and some forms of cancer (3, 4). Obesity is defined as systemic low grade inflammation (5). Some of the inflammatory cytokines, such as tumor necrosis factor-α(TNF-α), IL-1β, and IL-6, and C-reactive protein (CRP) are released from adipose tissue in obese animals. TNF-α not only promotes inflammation, but also induces insulin resistance by inhibiting insulin receptor activation. IL-1β, and IL-6, C-reactive protein also causes insulin resistance (6). In obesity animals, oxidative stress, which results from an imbalance between the amount of pro-oxidants and antioxidants, also contributes to a cause of insulin resistance (6). Leptin (LP) and adiponectin (ADN) are secreted from adipose tissue in obese animals. LP increases serum TNF-α and IL-6 concentrations and induces insulin resistance while ADN has anti-inflammatory properties and decreases the release of proinflammatory mediators (7).

High concentrations of circulating non-esterified fatty acid (NEFA) from accumulated visceral fat is one of characteristics of lipotoxicity (8, 9). As such, large amount of circulating NEFA from excessive accumulated visceral fat induces oxidative stress and inflammation (10, 11). In obese animals, β-oxidation of fatty acids in mitochondria is markedly activated in various tissues and excess amount of reactive oxygen species (ROS) is produced. Overproduced ROS is attributed to the one of pathogens for obesity and its associated inflammatory diseases (12). Consequently, some antioxidants and anti-inflammatory supplements are thought to be effective for ameliorating obesity conditions in animals as well as human (13–15).

Obesity is caused by over calorie intake and physical inactivity. Diet therapy is the most effective to prevent obesity in dogs and cats. Recently some phytogenics are reported to prevent high-fat diet-induced lipotoxicity in animals (16, 17). Such phytogenics have anti-oxidant and anti-inflammation effects (18, 19).

In the present study, we supplied obese dogs fed high-fat diet with plant mixture having anti-oxidant effect, and measured changes in concentrations of metabolites and hormones, and activities of enzymes in plasma of the dogs. The aim of this study is to discuss the effect of anti-oxidant supplement on lipid metabolism in obese animals.

## Materials and methods

### Animals

Twenty healthy Beagles [average age: 2 years (1–3 years), average body weight (BW):10.4 kg (9.4–11.3 kg), average body condition score (BCS) of five scale: 2.4 (2–3)] were enrolled in this study. Prior to the study, 20 healthy beagle dogs were kept in controlled condition and were fed on control diet (CD) for 2 months. Then, they were randomly divided into four groups. Ten dogs of control group were provided CD. For the five out of 10 CD dogs, 200 mg/ kg/day of anti-oxidant compound (Rv-PEM01-99, Kibun Foods, Inc., Tokyo, Japan) were supplemented. Another group of 10 dogs were provided high fat diet (HFD). For the five out of 10 dogs in HFD group, 200 mg/ kg/day of anti-oxidant compound (Rv-PEM01-99, Kibun Foods, Inc., Tokyo, Japan) were supplemented.

All the dogs were kept individually in cages measured 54 cm(height) × 45 cm (wide) × 72 cm (depth) and provided the same condition for 6 weeks at Narita Animal Science Laboratory Co., Ltd. (Narita, Japan), with the environment maintained at 24.0 ± 2.0°C and 55.0 ± 10.0% relative humidity, and on a 12:12 h, light: dark cycle (light on 8:00 a.m. to 8:00 p.m.).

### Induction of obesity with high-fat diet in dogs

HFD was designed by Nippon Pet Food, Co., Ltd. Differences in compounds between HFD and CD were as shown in Table 1. Calories in HFD were calculated as 403 kcal/100g food and those in CD food were calculated as 339 kcal/100 g food. HFD group dogs were given 38.8 kcal/kg /day of HFD, CD group dogs were given 30.8 kcal/kg/day of CD, respectively.

**Table 1:** Comparison of compounds between control and high fat diet.

	**Control diet**	**High fat diet**
Metabolic energy (kcal/100g)	339	403
Water (%)	8.7	6.1
Protein (%)	21.7	20.3
Lipid (%)	10.1	21.4
Linoleic acid (%)	1.72	2.55
Arachidonic acid (%)	ND	0.04
Decanoic acid (%)	ND	ND
Lauric acid (%)	ND	0.02
Myristic acid (%)	ND	0.39
Myristoleic acid (%)	ND	0.11
Pentadecanoic acid (%)	ND	0.04
Palmitic acid (%)	ND	5.37
Palmitoleic acid (%)	ND	0.58
Heptadecanoic acid (%)	ND	0.13
heptadecenoic acid (%)	ND	0.11
Stearic acid (%)	ND	2.42
Oleic acid (%)	ND	9.37
Linolenic acid (%)	ND	0.13
Arachidic acid (%)	ND	0.04
Icosenoic acid (%)	ND	0.09
Eicosapentaenoic acid (%)	ND	ND
Behenic acid (%)	ND	ND
Docosahexaenoic acid (%)	ND	ND
Crude fiber (%)	2.7	3.9
Nitrogen free extracts (%)	50.5	42.9
Ash (%)	6.3	5.4

*ND, not detected*.

### Supplementation with plant mixture

We used Rv-PEM01 prepared by Kibun Foods, Inc., Tokyo, Japan as antioxidant compound. Amount of each herb used for extraction (g) was as follows: *Rhus verniciflua* (90), *Ulmus hollandica* (60), *Polygonatum sibiricum* (50), *Lycium chinense* (10), *Ganoderma japonicum* (10), *Panax ginseng* (10) Total (230). Each of the six herbs was ground and added to the compound mixture (total volume is 230 g), which was then extracted with 10 vol of 70% ethanol in water at room temperature. The extracted solution was filtered, and the solvent was evaporated, filtered and lyophilized, yielding 60 g of lyophilized Rv-PEM01 from 230 g of herb powder (20). Urushiol, an allergenic substance found in *Rhus verniciflua* was completely removed. Two hundred milligrams of Rv-PEM01 is equivalent to 5 mg of quercetin derivative.

### Body weight (BW) and body conditioning score (BCS) measurement

BW and BCS were measured at the time of each blood sampling. Each subject was evaluated by the same veterinarian on-site, and classified by a 5-point scale system (1) Very thin, (2) Underweight, (3) Ideal, (4) Overweight, and (5) Obese, commonly used in Japan (21).

### Blood sampling

Five milliliters of each blood was collected from the jugular vein of each animal into the heparinized tubes before the experiment (0 week) and at the end of experiment (6 week).Blood collection was performed before the morning feeding and collected sample were immediately centrifuged at 2,000 x g for 10 min, 4°C. These samples were stored at −80°C until use.

### Metabolite, hormone, and enzyme analysis

Glucose (GLU), total cholesterol (TC), triglyceride (TG), total protein (TP), blood urea nitrogen (BUN), creatinine (CRE) concentrations and alanine aminotransferase (ALT), aspartate aminotransferase (AST), alkaline phosphatase (ALP), lactate dehydrogenase (LDH) activities were measured using an auto-analyzer (JCA-BM2250, JEOL Ltd., Tokyo, Japan) with the manufacture's reagents at FUJIFILM Monolith Co., Ltd (Tokyo, Japan). Plasma non-esterified fatty acid (NEFA) concentration was measured using a commercial kit, NEFA-C test (Wako Pure Chemical Industries, Inc., Tokyo Japan). Plasma insulin (INS), adiponectin (ADN), and tumor necrosis factor alpha (TNFα) were measured with Rat Insulin ELISA KIT(TMB) (AKRIN-010T, Shibayagi Co., Gumma, Japan), mouse/rat adiponectin ELISA kit (Otsuka Pharmaceutical Co., Ltd., Tokyo, Japan), TNF Alpha Dog Elisa kit (LS-F1347-1, Life Span Bioscience, Inc, Seattle, USA), respectively.

Leucocytic AMP-activated protein kinase (AMPK) activity was measured with a commercial ELISA kits, CycLex AMPK Kinase kit (CycLex Co., Ltd. Nagano, Japan).

The AMPK activity was measured at 30°C for 30min and was expressed as ng of phosphorylated substrates per minute per mg of protein (specific activity).

Protein concentration in white blood cell cytosol fraction was measured with the Bradford method using bovine serum albumin as the standard (22).

### Statistics

All values are expressed as mean ± standard error (SE). Statistical significance was determined by paired-*t* test. The significance level was set at *p* < 0.05.

## Results

### Changes in body weight (BW)

Changes in mean BW (kg) ±SE are shown in Tables 2, 3. At the end of the experiment (6 weeks), animals of control diet (CD) group, high fat diet (HFD) group, CD+Rv-PEM01-99 group (CD+Rv-PEM01-99) and HFD+Rv-PEM01-99 group (HDF+Rv-PEM01-99) showed 7.6, 23, 5.8, 22.9% of BW increase compared to the pre-experiment (0 week) BW, respectively. Compared to CD group, CD+Rv-PEM 01-99 group showed fewer increase in BW (5.8-percent gain) than CD group (7.6-percent gain) while HFD+Rv-PEM 01-99 group showed no changes in BW compared to HFD group over the 6 weeks of the experiment. Significant changes in BCS of all groups were not observed during the experiment.

**Table 2:** Changes in biomarker levels in obese dogs fed with high fat diet.

	**Without Rv-PEM01-99**		**With Rv-PEM01-99**	
	**0 W**	**6 W**	**0 W**	**6 W**
BW (Kg)	10.4 ± 0.3	12.8 ± 0.4	10.5 ± 0.3	12.9 ± 0.4
INS (ng/mL)	0.3 ± 0.1	0.6 ± 0.2	0.1 ± 0.0	0.4 ± 0.1
Adipo (μg/mL)	27.9 ± 6.5	38.8 ± 7.8	21.3 ± 2.8	25.9 ± 3.9
AMPK (ng/mg protein min)	8.7 ± 0.8(3)	13.2 ± 0.9	12.7 ± 2.5(4)	13.7 ± 2.6
TNFα (pg/mL)	0.1 ± 0.0	0.1 ± 0.0	0.1 ± 0.0	0.1 ± 0.0
NEFA (mEq/L)	0.69 ± 0.05	0.61 ± 0.12	0.60 ± 0.07	0.60 ± 0.12
TG (mg/dL)	36.0 ± 1.2	78.8 ± 17.2	32.4 ± 3.7	59.2 ± 14.9
MDA (μmol/L)	1.14 ± 0.10	2.00 ± 0.40	1.39 ± 0.11	1.60 ± 0.29
TC (mg/dL)	120.4 ± 11.0	199.6 ± 29.5^*b^	139.2 ± 14.1	181.0 ± 10.6^*a^
AST (IU/L)	26.8 ± 0.4	32.4 ± 2.7	25.6 ± 0.4	32.8 ± 2.6
ALT (IU/L)	40.0 ± 2.4	33.0 ± 2.2	42.0 ± 2.7	41.4 ± 4.7
ALP (IU/L)	154.6 ± 16.6	141.2 ± 15.6	156.0 ± 13.7	85.2 ± 4.2^*a^
LDH (IU/L)	88.6 ± 12.8	82.4 ± 8.2	71.6 ± 10.5	70.6 ± 2.7
TP (g/dL)	6.7 ± 0.1	7.2 ± 0.2^*b^	6.4 ± 0.1	6.6 ± 0.1
BUN (mg/dL)	12.6 ± 0.7	17.4 ± 1.2^*b^	13.8 ± 0.6	12.2 ± 1.7
CRE (mg/dL)	0.7 ± 0.0	0.7 ± 0.0	0.7 ± 0.0	0.7 ± 0.0
GLU (mg/dL)	91.6 ± 2.9	93.6 ± 1.4	91.6 ± 3.1	94.8 ± 3.0

*Data are presented as mean ± SE*.

*The numbers in parenthese indicate the number of animals examined*.

*a *Significant (p < 0.05) when compared against 0 W of High Fat Diet +Rv-PEM01-99group (paired-T test)*.

*b *Significant (p < 0.05) when compared against 0 W of High Fat Diet group (paired -T test)*.

**Table 3:** Changes in biomarker levels in control dogs fed with control diet.

	**Without Rv-PEM01-99**		**With Rv-PEM01-99**	
	**0 W**	**6 W**	**0 W**	**6 W**
BW (Kg)	10.5 ± 0.2	11.3 ± 0.3	10.3 ± 0.3	10.9 ± 0.5
INS (ng/mL)	0.2 ± 0.1	0.3 ± 0.1	0.2 ± 0.0	0.3 ± 0.0
Adipo (μg/mL)	18.4 ± 4.4(4)	27.5 ± 3.7	28.3 ± 6.8	30.9 ± 4.2
AMPK (ng/mg protein min)	6.8 ± 1.3	11.1 ± 1.5	9.5 ± 4.8(4)	11.5 ± 1.2
TNFα (pg/mL)	0.1 ± 0.0	0.1 ± 0.0	0.1 ± 0.0	0.1 ± 0.0
NEFA (mEq/L)	0.73 ± 0.08	0.68 ± 0.12	0.83 ± 0.17	0.95 ± 0.18
TG (mg/dL)	30.6 ± 1.4	38.0 ± 7.4	40.2 ± 3.5	25.4 ± 3.5^*a^
MDA (μmol/L)	1.47 ± 0.19	1.46 ± 0.10	1.49 ± 0.14	0.98 ± 0.03^*a^
TC (mg/dL)	132.8 ± 3.7(4)	131.6 ± 10.0	118.8 ± 7.1	133.2 ± 6.8
AST (IU/L)	28.8 ± 0.4	35.2 ± 2.7	26.4 ± 0.4	35.8 ± 1.4
ALT (IU/L)	40.5 ± 2.9	30.2 ± 5.9	44.4 ± 4.4	42.0 ± 2.4
ALP (IU/L)	152.0 ± 4.3	166.6 ± 40.2	154.4 ± 4.4	160.8 ± 26.4
LDH (IU/L)	83.4 ± 6.3	113.6 ± 14.1	95.6 ± 7.6	60.8 ± 6.0^*1^
TP (g/dL)	7.2 ± 0.1	7.0 ± 0.2	6.4 ± 0.0	6.4 ± 0.0
BUN (mg/dL)	12.8 ± 0.7	12.0 ± 0.8	14.6 ± 0.2	13.8 ± 1.5
CRE (mg/dL)	0.7 ± 0.0	0.7 ± 0.0	0.7 ± 0.0	0.7 ± 0.0
GLU (mg/dL)	85.0 ± 1.4	79.8 ± 2.4	98.4 ± 2.7	95.6 ± 2.2

*Data are presented as mean ± SE*.

*The numbers in parenthese indicate the number of animals examined*.

*a *Significant (p < 0.05) when compared against 0 W of Control Diet +Rv-PEM01-99group (paired-T test)*.

### Changes in plasma metabolites

Over the 6 weeks of the experiment, dogs in CD+Rv-PEM01-99 group showed significantly decreased TG concentrations (paired-*t, p* < 0.05) while dogs in HFD+Rv-PEM01-99 group showed increased TG concentrations. Plasma MDA levels in CD+Rv-PEM 01-99 group were significantly decreased (MDA: 0.98 ± 0.03 μmol/L). Although obese dogs in HFD group showed significantly increased BUN concentrations, obese dogs in HFD+Rv-PEM01-99 group showed decreased BUN concentrations at the end of the experiment. Plasma TNF-α activities in all group were not observed during the experiment. Plasma NEFA levels in all groups were increased within the healthy normal ranges.

### Changes in plasma hormones

Although not significant, ADN concentrations in obese dogs fed HFD group were increased compared to those in control dogs at the end of the experiment. Plasma INS and ADN concentrations in all groups increased within the healthy normal ranges as AMPK activities in WBC cytosolic increased.

### Changes in plasma enzymes

At the end of experiment, LDH activities in CD group were increased. However, LDH activities in control dogs supplemented with Rv-PEM 01-99 were significantly decreased (60.8 ± 6.0 IU/L). Significant changes in LDH activities in obese dogs were not observed.

## Discussion

In dogs and cats, the availability of functional food for weight loss, against behavioral disorders, and chronic inflammation, has been discussed by many researchers (23–28), and the role of supplements with anti-oxidant and anti-inflamatory effect has been reported (29, 30) There are many reports discussing anti-obesity effects of anti-oxidant polyphenols such as isoflavone (31–33) anthocyanin (34–36), catechin (37–39), and quercetin (40–42). Especially, quercetin glycoside is a well-known compound since its metabolic pathway route in human intestine is clarified (43). Yoshimura et al. demonstrated body fat reducing effect of quercetin glycosides on obese humans (44, 45). In our preliminary study, we observed BW decrease in mouse fed High fat +2%Rv-PEM 01-99 for 60 days. To investigate anti-obesity effect of Rv-PEM 01-99 in dogs, we developed obese dogs by feeding HFD for 6 weeks.

At the end of experiment, obese dogs fed HFD showed 23% BW increase and increased TC, TP, BUN concentrations. Although many researcher reported decreased serum/plasma ADN concentrations in obese animals (5, 46–48), plasma INS and ADN concentrations were increased in obese dogs fed HFD. Furthermore, obese dogs fed HFD seemed to show no inflammation since changes in plasma TNF-αlevels were not observed. The activities of AMPK, an energy metabolic parameter, in WBC cytosol, were increased accompanied by plasma ADN increase. As we have previously proposed in cats (49, 50), the above mentioned conditions indicate no pathology, but a healthy state seen at an early stage of weight gain in dogs.

As shown in Table 3, since plasma TG and MDA levels, LDH activities were significantly decreased at the end of experiment in control dogs supplemented Rv-PEM 01-99, anti-oxidant effect and improvement effect of liver function and lipid metabolism are expected in healthy dogs by use of Rv-PEM 01-99.

In the present study, anti-obesity mechanism of Rv-PEM 01-99 in dogs was not clear.

As shown in Table 1, high fat food we used was specially designed to increase canine body weight by adding saturated fatty acid such as palmitic acid, stearic acid which contain in beef fallow, while control food we used was a commercial diet and was designed to meet the amount of lipid and linoleic acid for canine growth requirement. From our data as shown in Tables 2, 3, we did not induce pathological obese condition by feeding HF food for 6 weeks. Therefore, it is thought that anti-obesity efffect of Rv-PEM 01-99 in dogs was not clear.

Yoshimura et al. (44, 45) administered 110–275 mg of enzymatically modified isoquercitrin for 12 weeks to human to investigate anti-obesity effect. *Rhus verniciflua* leaf contains physiologicall active substances such as fustin, fisetin, sulfuretin, qurercetin, and butein. (51). It was demonstrated that quercetin showed anti-oxidant (52), anti-inflammatory (53), anti-arteriosclerosis (54), anti-tumor (20, 55–57), anti-hypertension (58), and vascular atony effects (59). Simizu et al. have demonstrated the regulatory mechanism of apolipoprotein gene transcription by quercetin on the inhibition of cylomicron synthesis in human small intestine (60).

This study is a preliminary investigation. To discuss the effectiveness of Rv-PEM 01-99 for obese dogs, we have to design new experiment (1) enrolling many dogs, (2) using larger amounts of Rv-PEM 01-99, and (3) taking longer experimental period since Christmann et al. admitted effectiveness of weight management/loss food by use of 38–162 dogs for 6 months (24, 27).

## Ethics statement

Ethical approval for this study was from Narita Animal Science Laboratory Co., Ltd. Research Animal Ethical Committee (17-C042).

## Author contributions

TA conceived and designed the study. KKaw collected data with TMu and TMi. YO and IY performed critical analysis and interpretation of data with KKaw. KS and KKad designed Rv-PEM01-99. KKaw and YO wrote the manuscript.

### Conflict of interest statement

KS and KK were employed by company Kibun Foods Inc. The remaining authors declare that the research was conducted in the absence of any commercial or financial relationships that could be construed as a potential conflict of interest.

## Abbreviations

ADN: adiponectin
AMPK: AMP-activated protein kinase
ALP: alkaline phosphatase
ALT: alanine aminotransferase
AST: aspartate aminotransferase
BCS: body condition score
BUN: blood urea nitrogen
BW: body weight
CD: control diet
CRE: creatinine
GLU: glucose
HFD: high fat diet
INS: insulin
LDH: lactate dehydrogenase
LP: leptin
MDA: malondialdehyde
NEFA: non-esterified fatty acid
SE: standard error
TC: total cholesterol
TP: total protein
TG: triglyceride TNF-α; tumor necrosis factor alpha.

## References

[1] Lund EM Armstrong PJ Kirk CA Klausner JS Prevalence and risk factors for obesity in adult dogs from private US veterinary practices. Int J Appl Res Vet Med. (2006) 4:177–86. Available online at: http://citeseerx.ist.psu.edu/viewdoc/download?doi=10.1.1.467.885&rep=rep1&type=pdf

[2] Chandler M Cunningham S Lund EM Khanna C Naramore R Patel A. Obesity and associated comorbidities in people and companion animals: a one health perspective. J Comp Pathol. (2017) 156:296–309. 10.1016/j.jcpa.2017.03.006 28460795

[3] de Godoy MRC and Swanson KS. Companion animals symposium: nutrigenomics: using gene expression and molecular biology data to understand pet obesity. J Anim Sci. (2013) 91:2949–64. 10.2527/jas2012-5860 23296821

[4] Takahara M Shimomura I. Metabolic syndrome and lifestyle modification. Rev Endocr Metab Disord. (2014) 15:317–27. 10.1007/s11154-014-9298-8 25263290

[5] German AJ Ryan VH German AC Wood IS Trayhurn P. Obesity, its associated disorders and the role of inflammatory adipokines in companion animals. Vet J. (2010) 185:4–9. 10.1016/j.tvjl.2010.04.004 20472476

[6] Laflamme DP. Companion animals symposium: obesity in dogs and cats: what is wrong with being fat? J Anim Sci. (2012) 90:1653–62. 10.2527/jas2011-4571 21984724

[7] Peng CH Lin HT Chung DJ Huang CN Wang CJ. Mulberry leaf extracts prevent obesity-induced NAFLD with regulating adipocytokines, inflammation and oxidative stress. J Food Drug Anal. (2018) 26:778–87. 10.1016/j.jfda.2017.10.008 29567249 PMC9322217

[8] Unger RH. Lipid overload and overflow: metabolic trauma and the metabolic syndrome. Trends Endocrinol Metab. (2003) 14:398–403. 10.1016/j.tem.2003.09.008 14580758

[9] DeFronzo RA. Insulin resistance, lipotoxicity, type 2 diabetes and atherosclerosis: the missing links. The Claude Bernard Lecture 2009. Diabetologia (2010) 53:1270–87. 10.1007/s00125-010-1684-1 20361178 PMC2877338

[10] Shi X Li D Deng Q Li Y Sun G Yuan X. NEFAs activate the oxidative stress-mediated NF-kB signaling pathway to induce inflammatory response in calf hepatocytes.J Steroid Biochem Mol Biol. (2015) 145:103–12. 10.1016/j.jsbmb2014.10.014 25465477

[11] Medina-Luna D Santamaria-Olmedo MG Zamuido-Cuevas Y Martinez-Flores K Fernandez-Torres J Martinez-Nava GA. Hyperlipidemic microenvironment conditionates damage mechanisms in human chondrocytes by oxidative stress. Lipid Health Dis. (2017) 16:114. 10.1186/s12944-017-0510-x 28606092 PMC5468939

[12] Natori R Fernando N Dahlenburg T Jiao H Aggio-Bruce R Barnett N. Obesity-induced metabolic disturbance drives oxidative stress and complement activation in the retinal environment. Mol Vis. (2018) 24:201–17. 29527116 PMC5842320

[13] Ribeiro MR Lima RPA Lisboa JVC Chaves TR Luna RCP do Nascimento RAF. Influence of the C677T polymorphism of the MTHFR gene on oxidative stress in women with overweight or obesity: response to dietary folate intervention. J Am Coll Nutr. (2018) 27:1–8. 10.1080/07315724.2018.1460224 29702041

[14] Kim K Chung MH Park S Cha J Baek JH Lee SY. ER stress attenuation by aloe-derived polysaccharides in the protection of pancreatic β-cells from free fatty acid-induced lipotoxicity. Biochem Biophys Res Commun. (2018) 500:797–803. 10.1016/j.bbrc.2018.04.162 29684344

[15] Otton R Bolin AP Ferreira LT Marinovic MP Rocha ALS Mori MA. Polyphenol-rich green tea extract improves adipose tissue metabolism by down-regulation miR-335 expression and mitigating insulin resistance and inflammation. J Nutr Biochem. (2018) 57:170–9. 10.1016/j.jnutbio.2018.03.024 29734116

[16] Mahmoudi M Charradi K Limam F Aouani E. Grape seed and skin extract as an adjunct to Xenical therapy reduces obesity, brain lipotoxicity and oxidative stress in high fat diet fed rats. Obes Res Clin Pract. (2018) 12:115–26. 10.1016/j.orcp.2016.04.006 27161420

[17] Bedhiafi T Charradi K Ben Azaiz M Mahmoudi M Msakni I Jebari K. Supplementation of grape seed and skin extract to orlistat therapy prevents high-fat diet-induced murine spleen lipotoxicity. Appl Physiol Nutr Metab. (2018) 43:782–94. 10.1139/apnm-2017-0743 29514007

[18] Jiao R Liu Y Gao H Xiao J So KF. The Anti-oxidant and antitumor properties of plant polysaccharides. Am J Chin Med. (2016) 44:463–88. 10.1142/S0192415X16500269 27109156

[19] Varga E Domokos E Fogarasi E Steanesu R Fulop I Croitoru MD. Polyphenolic compounds analysis and antioxidant activity in fruit of *Prunus spinose* L. Acta Pharm Hung. (2017) 87:19–25. 29489094

[20] Hiruma W Suruga K Kadokura K Tomita T Miyata A Sekino Y Antitumor effects and acute oral toxicity studies of a plant extract mixture containing *Rhus verniciflua* and some other herbs. Open J Immunol. (2015) 5:39–49. 10.4236/oji.2015.51005

[21] Mori N Lee P Kondo K Saito T Arai T. Potential use of cholesterol lipoprotein profile to confirm obesity status in dogs. Vet Res Commun. (2011) 35:223–35. 10.1007/s11259-011-9466-x 21327518

[22] Bradford MM. A rapid and sensitive method for the quantitation of microgram quantities of protein utilizing the principle of protein dye binding. Anal Biochem. (1976) 72:248–54. 10.1016/0003-2697(76)90527-3 942051

[23] Towll TL Forrester SD Cross S Tolsdorf G Bernat S Roth S Evaluation of a weight management food designed to increase basal metabolism in a home setting. Int J Appl Res Vet Med. (2015) 13:14–22. Available online at: http://www.jarvm.com/articles/Vol13Iss1/Vol12%20Iss3Towell.pdf#search='Evaluation+of+a+weight+management+food+designed+to+increase+basal+metabolism+in+a+home+setting.+Intern+J+Apple+Res+Vet+Med+%282015%29+13%281%29%3A+1422

[24] Christmann U Becvarova I Werre S Meyer HP Effectiveness of a new weight management food to achieve weight loss and maintenance in client-owned obese dogs. Int J Appl Res Vet Med. (2015) 13:104–16. Available online at: https://www.researchgate.net/publication/282712703_Effectiveness_of_a_new_weight_management_food_to_achieve_weight_loss_and_maintenance_in_client-owned_obese_dogs 10.1177/1098612X15599823 PMC11112234 26303604

[25] Di Cerbo A Centenaro S Beribe F Laus F Cerquetella M Spanterna A. Clinical evaluation of an anti-inflammatory and antitoxidant diet in 30 dogs affected by chronic otitis externa: preliminary results. Vet Res Commun. (2016) 40:29–38. 10.1007/s11259-015-9651-4 26743397 PMC4754334

[26] Sechi S Di Cerbo A Canello S Guidetti G Chiavolelli F Fiore E. Effects in dogs with behavioural disorders of a commercial natraceutical diet on stress and neuroendocrine parameters. Vet Rec. (2016) 180:18. 10.1136/vr.103865 27885066 PMC5284471

[27] Christmann U Becvarova I Were S Meyer HP Effectiveness of a new dietetic food to achieve weight loss and to improve mobility in client-owned obese dogs with osteoarthritis. Int J Appl Res Vet Med. (2018) 16:81–93. Available online at: https://www.jarvm.com/articles/Vol16Iss1/Vol16%20Iss1%20Christman.pdf#search='Christmann+U%2C+Becvarova+I%2C+Were+S%2C+Meyer+HP.+Effectiveness+of+a+new+weight+management+food+to+achieve+weight+loss+and+maintenance+in+clientowned+obese+dogs.+Intern+J

[28] Di Cerbo A Morales-Medina JC Palmieri B Pezzuto F Cocco R Flores G. Functional foods in pet nutrition: foucus on dogs and cats. Res Vet Sci. (2017) 112:161–6. 10.1016/j.rvsc.2017.03.020 28433933

[29] Sechi S Chiavolelli F Spissu N Di Cerbo A Canello S Guidetti G. An antioxidant dietary supplement improves brain-derived neurotrophic factor levels in serum of aged dogs: preliminary results. J Vet Med. (2015) 2015:412501. 10.1155/2015/412501 26464952 PMC4590864

[30] Guidetti G Di Cerbo A Giovazzino A Rubino V Palatucci AT Centenaro S. *In vitro* effects of some botanicals with anti-inflammatory and antitoxic activity. J Immunol Res. (2016) 2016:5457010. 10.1155/2016/5457010 27597982 PMC5002466

[31] Yao Y Li XB Zhao W Zeng YY Shen H Xiang H. Anti-obesity effect of an isoflavone fatty acid ester on obese mice induced by high fat diet and its potential mechanism. Lipids Health Dis. (2010) 9:49. 10.1186/1476-511X-9-49 20482839 PMC2889992

[32] Ahn TG Yang G Lee HM Kim MD Choi HY Park KS. Molecular mechanisms underlying the anti-obesity potential of prunetin, an O-methylated isoflavone. Biochem Pharmacol. (2013) 85(10):1525–33. 10.1016/j.bcp.2013.02.020 23438470

[33] Jo YH Choi KM Liu Q Kim SB Ji HJ Kim M. Ant-obesity effect of 6,8-Diprenylgenistein, an isoflavonoid of *Cudrania tricuspidata* fruits in high-fat diet-induced obese mice. Nutrients (2015) 7:10480–90. 10.3390/nu7125544 26694457 PMC4690096

[34] Kwon SH Ahn IS Kim SO Kong CS Chung HY Do MS. Anti-obesity and hypolipidemic effects of black soybean anthocyanins. J Med Food (2007) 10:552–6. 10.1089/jmf.2006.147 17887951

[35] Wu T Yu Z Tang Q Song H Gao Z Chen W. Honeysuckle anthocyanin supplementation prevents diet-induced obesity in C57BL/6 mice. Food Funct. (2013) 4:1654–61. 10.1039/c3fo60251f 24081320

[36] Ju R Zheng S Luo H Wang C Duan L Sheng Y. Purple sweet potato attenuate weight gain in high fat diet induced obese mice. J Food Sci. (2017) 82:787–93. 10.1111/1750-3841.13617 28135399

[37] Marrelli M Loizzo MR Nicoletti M Menichini F Conforti F. *In vitro* investigation of the potential health benefits of wild Mediterranean dietary plants as anti-obesity agents with α-amylase and pancreatic lipase inhibitory activities. J Sci Agric. (2014) 94:2217–24. 10.1002/jsfa.6544 24535986

[38] Mielgo-Ayuso J Barrenechea L Alcorta P Larrarte E Margareto J Labayen I. Effects of dietary supplementation with epigallocatechin-3-gallate on weight loss, energy homeostasis, cardiometabolic risk factors and liver function in obese women: randomized, double-blind, placebo-controlled clinical trial. Br J Nutr. (2014) 111:1263–71. 10.1017/S0007114513003784 24299662

[39] Rahman HA Sahib NG Saari N Abas F Ismail A Mumtaz MW. Anti-obesity effect of ethanolic extract from Cosmos caudatus Kunth leaf in lean rats fed a high fat diet. BMC Complement Altern Med. (2017) 17:122. 10.1186/s12906-017-1640-4 28228098 PMC5322639

[40] Hoek-van den Hil EF van Schothorst EM van der Stelt I Swarts HJ Venema D Sailer M. Quercetin decreases high-fat diet induced body weight gain and accumulation of hepatic and circulating lipids in mice. Genes Nutr. (2014) 9:418. 10.1007/s12263-014-0418-2 25047408 PMC4172648

[41] Dong J Zhang X Zhang L Bian HX Xu N Bao B. Quercetin reduces obesity-associated ATM infiltration and inflammation in mice: a mechanism including AMPKα1/ SIRT1. J Lipid Res. (2014) 55:363–74. 10.1194/jlr.M038786 24465016 PMC3934722

[42] Seo MJ Lee YJ Hwang JH Kim KJ Lee BY. The inhibitory effects of quercetin on obesity and obesity-induced inflammation by regulation of MAPK signaling. J Nutr Biochem. (2015) 26:1308–16. 10.1016/j.jnutbio.2015.06.005 26277481

[43] Murota K Terao J. Antioxidative flavonoid quercetin: implication of its intestinal absorption and metabolism. Arch Biochem Biophys. (2003) 417:12–7. 10.1016/S0003-9861(03)00284-4 12921774

[44] Yoshimura M Maeda A Takehara I Abe K Ohta H Kiso Y Body fat reducing effect and safety of the beverage containing polyphenols derived from Japanese pagoda tree (enzymatically modified isoquercitrin) in overweight and obese subjects. Jpn Pharmacol Ther. (2008) 36:919–30. Available online at: http://lifescience.co.jp/yk/yk08/oct/ab4.html

[45] Yoshimura M Maeda A Nakamura J Kitagawa Y Shibata H Fukuhara I Body fat reducing effect of continuous consumption of the beverage containing quercetin glucosides (enzymatically modified isoquercitrin) in obese subjects. Jpn Pharmacol Ther. (2012) 40:901–14. Available online at: http://www.lifescience.co.jp/yk/yk12/oct/ab8.html

[46] Ishioka K Omachi A Sagawa M Shibata H Honjoh T Kimura K. Canine adiponectin: cDNA structure, mRNA expression in adipose tissues and reduced plasma levels in obesity. Res Vet Sci. (2005) 80:127–32. 10.1016/j.rvsc.2005.05.011 16051287

[47] Radin MJ Sharkey LC Holycross BJ. Adipokines: a review of biological and analytical principles and an update in dogs, cats, and horses. Vet Clin Pathol. (2009) 38:136–56. 10.1111/j.1939-165X.2009.00133.x 19392760

[48] Zoran DL. Obesity in dogs and cats: a metabolic and endocrine disorder. Vet Clin North Small Anim Pract. (2010) 40(2):221–39. 10.1016/j.cvsm.2009.10.009 20219485

[49] Kawasumi K Iwazaki E Okada Y Arai T. Effectiveness of feline body mass index (fBMI) as new diagnostic tool for obesity. JJVR (2016) 64:51–6. 10.14943/jjvr.64.1.51 27348888

[50] Okada Y Kobayashi M Sawamura M Arai T. Comparison of visceral fat accumulation and metabolome markers among cats of varying BCS and novel classification of feline obesity and metabolic syndrome. Front Vet Sci. (2017) 4:17. 10.3389/fvets.2017.00017 28261588 PMC5306360

[51] Kim S Park SE Sapkota K Kim MK Kim SJ. Leaf extract of *Rhus verniciflua* strokes protects dopaminergic neuronal cells in a rotenone model of Parkinson's disease. J Pharm Pharmacol. (2011) 63:1358–67. 10.1111/j.2042-7158.2011.01342.x 21899552

[52] Forte L Torricelli P Boanini E Rubini K Fini M Bigi A. Quercetin and alendronate multifunctionalized materials as tools to hinder oxidative stress damage. J Biomed Mater Res A (2017) 105:3293–303. 10.1002/jbm.a.36192 28865182

[53] Guazalli CFS Staurengo-Ferrari L Zarpelon AC Pinho-Riberio FA Ruiz-Miyazawa KW Vicentini FTMC. Quercetin attenuates zymosan-induced arthritis in mice. Biomed Pharmacother. (2018) 102:175–84. 10.1016/j.biopha.2018.03.057 29554596

[54] Loke WM Proudfoot JM Hodgson JM McKinley AJ Hime N Magat M. Specific dietary polyphenols attenuate atherosclerosis in apolipoprotein E-knockout mice by alleviating inflammation and endothelial dysfunction. Arterioscler Thromb Vasc Biol. (2010) 30:749–57. 10.1161/ATVBAHA 20093625

[55] Suruga T Hiruma W Kadokura K Tomita T Miyata A Sekino Y Antitumor and apoptopic effects of a plant exact mixture containing *Rhus verniciflua* and other herbs in human leukemia cells. Drug Res. (2017) 67:127–30. 10.1055/s-0042-113458 27626609

[56] Li S Yuan S Zhao Q Wang B Wang X Li K. Quercetin enhances chemotherapeutic effect of doxorubicin against human breast cancer cells while reducing toxic side effects of it. Bimed Pharmacother. (2018) 100:441–7. 10.1016/j.biopha.2018.02.055 29475141

[57] Chan ST Chuang CH Lin YC Liao JW Lii CK Yeh SL. Quercetin enhances the antitumor effect of trichostatin A and suppresses muscle wasting in tumor-bearing mice. Food Funct. (2018) 9:871–9. 10.101039/c7fo01444a 29292417

[58] Duarte J Perez-Palencia R Vargas F Ocete MA Perez-Vizcaino F Zarzuelo A. Antihypertensive effects of the flavonopid quercetin in spontaneously hypertensive rats. Br J Pharmacol. (2001) 133:117–24. 10.1038/sj.bjp.0704064 11325801 PMC1572775

[59] Perez-Vizcaino F Ibarra M Cogolludo AL Duarte J Zaragoza-Arnaez F Moreno L. Endothelium-independent vasodilator effects of the flavonoid quercetin and its methylated metabolites in rat conductance and resistance arteries. J Pharmacol Exp Ther. (2002) 302:66–72. 10.1124/jpet.302.1.66 12065701

[60] Shimizu M Li J Inoue J Sato R. Quercetin represses apolipoprotein B expression by inhibiting the transcriptional activity of C/EBPβ. PLoS ONE (2015) 10:e0121784. 10.1371/journal.pone.0121784 25875015 PMC4398426

